# The ratio of serum n-3 to n-6 polyunsaturated fatty acids is associated with diabetes mellitus in patients with prior myocardial infarction: a multicenter cross-sectional study

**DOI:** 10.1186/s12872-017-0479-4

**Published:** 2017-01-26

**Authors:** Masao Takahashi, Jiro Ando, Kazunori Shimada, Yuji Nishizaki, Shigemasa Tani, Takayuki Ogawa, Masato Yamamoto, Ken Nagao, Atsushi Hirayama, Michihiro Yoshimura, Hiroyuki Daida, Ryozo Nagai, Issei Komuro

**Affiliations:** 1 Department of Cardiovascular Medicine, Graduate School of Medicine, The University of Tokyo, Hongo 7-3-1, Bunkyo-ku, Tokyo, 113-8655 Japan; 2 Department of Cardiology, Juntendo University Graduate School of Medicine, Hongo 3-1-3, Bunkyo-ku, Tokyo, 113-8431 Japan; 3 Department of Cardiology, Nihon University Hospital, 1-6 Kanda Surugadai Chiyoda-ku, Tokyo, 101-8309 Japan; 4 Divison of Cardiology, Department of Internal Medicine, The Jikei University School of Medicine, Nishishinbashi 3-19-18, Minato-ku, Tokyo, 105-8471 Japan; 5 Department of Cardiology, Sempo Takanawa Hospital, Takanawa 3-10-11, Minato-ku, Tokyo, 108-8606 Japan; 6 Division of Cardiology, Department of Medicine, Nihon University School of Medicine, 30-1Ohyaguchi Kamichou Itabashi-ku, Tokyo, 173-8610 Japan; 7 Jichi Medical University, Yakushiji 3311-159, Shimotsuke city, Tochigi 329-0498 Japan

**Keywords:** Polyunsaturated fatty acids (PUFAs), Prior myocardial infarction (PMI), Diabetes mellitus (DM), High-sensitivity C-reactive protein (hs-CRP), Eicosapentaenoic acid (EPA), Inflammation, Statin

## Abstract

**Background:**

In prior myocardial infarction (PMI) patients, diabetes mellitus (DM), dyslipidemia, and hypertension increase the risk of secondary cardiovascular events. Although a decreased ratio of serum eicosapentaenoic acid (EPA) to arachidonic acid (AA; EPA/AA) has been shown to significantly correlate with the onset of acute coronary syndrome, the associations between polyunsaturated fatty acid (PUFA) levels and coronary risk factors in PMI patients have not been evaluated thoroughly. This study aimed to assess the associations between PUFAs levels and the risk factors in PMI patients.

**Methods:**

We enrolled 1733 patients with known PUFA levels who were treated in five divisions of cardiology in a metropolitan area of Japan, including 303 patients with PMI. EPA/AA and docosahexaenoic acid (DHA) to AA level ratio (DHA/AA) in patients with and without PMI were analyzed according to presence of coronary risk factors.

**Results:**

Diabetes patients with PMI had significantly lower EPA/AA and DHA/AA than diabetes patients without PMI (EPA/AA: *P* <0.01; DHA/AA: *P* =0.003), with no such differences in dyslipidemia and hypertension patients. In DM patients with high high-sensitivity C-reactive protein (hs-CRP) levels (>0.1 mg/dL), EPA/AA was low in individuals who also had PMI, whereas DHA/AA was not (EPA/AA, with PMI: 0.43 ± 0.24; without PMI: 0.53 ± 0.30, *P* < 0.05). Moreover, patients on statins had significantly lower DHA/AA ratios, whereas the EPA/AA ratio did not depend on statin use. Multiple regression analysis revealed that statin use in DM patients was associated with low DHA/AA but not EPA/AA.

**Conclusion:**

PMI patients with DM have low EPA/AA and DHA/AA. EPA/AA and DHA/AA are differently related to hs-CRP level in DM patients with PMI. Statin use can potentially affect DHA/AA but not EPA/AA, and therefore EPA/AA ratio is a better marker of assessment for cardiovascular events.

**Electronic supplementary material:**

The online version of this article (doi:10.1186/s12872-017-0479-4) contains supplementary material, which is available to authorized users.

## Background

Much evidence has been accumulated indicating that patients with prior myocardial infarction (PMI) have a greater chance of developing additional cardiac events, necessitating strict management for secondary prevention [1, 2]. Several clinical studies have reported that n-3 polyunsaturated fatty acids (PUFAs), including eicosapentaenoic acid (EPA) and docosahexaenoic acid (DHA), reduce the risk of secondary cardiovascular events [3–5]. The JELIS study demonstrated that adding EPA to statin therapy significantly reduced major coronary events compared with statin therapy alone in dyslipidemia patients [6]. It is well known that n-3 PUFAs have anti-inflammatory effects, such as reduction of production of inflammatory eicosanoids, cytokines, and reactive oxygen species and stimulation of expression of adhesion molecules, whereas arachidonic acid (AA), which gives rise to many inflammatory eicosanoids (e.g., thromboxaneA2), plays a central role in inflammation related to injury and in many disease mechanisms [7]. Patients with cardiovascular disease may benefit from n-3 PUFA supplementation given its anti-inflammatory effect. However, a recent meta-analysis of randomized controlled trials of n-3 PUFAs intake failed to demonstrate a preventive effect with regard to cardiovascular events [8]. One possible reason for this lack of effect is improvements in other cardioprotective therapies, such as statin use [9, 10]. Another reason could be the relatively low dose of n-3 PUFAs compared to that in the JELIS study [9]. Overall, much controversy exists over the use of n-3 PUFAs for secondary prevention. It has been reported that reduced serum levels of n-3 PUFAs are associated with an increased incidence of cardiovascular events and mortality [11, 12]. Moreover, a decreased ratio of serum EPA to AA (EPA/AA) has been shown to significantly correlate with onset of acute coronary syndrome [13] and with a high prevalence of complex coronary lesions [14]. Low EPA/AA and DHA/AA ratios can be potentially used to identify patients in need of n-3 PUFAs supplementation. It is also feasible to assess the EPA/AA and DHA/AA ratios, which have been reported to be markers of cardiovascular events [13, 14] because EPA and AA are competing substrates of cyclooxygenase enzymes [15]. Therefore, the ratios of EPA and DHA to AA may reflect cardiovascular inflammation in patients with cardiovascular diseases, including PMI. Furthermore, because previous studies indicate a strong relationship between inflammation and cardiovascular risk [16, 17], the association between serum PUFA levels and inflammation should be evaluated.

The aim of the present study was to determine the serum levels of n-3 and n-6 PUFAs in patients with PMI as well as to identify factors associated with low EPA/AA and DHA/AA ratios.

## Methods

### Study design and patient enrollment

This was a multicenter observational study performed at five centers (four university hospitals and one community hospital) located in Tokyo. We enrolled 1733 Japanese patients who were treated in the divisions of cardiology at these five centers from January 2004 to May 2011. This cohort included 303 patients with PMI. Serum PUFA levels were determined in all of these patients. Patients were excluded if they were receiving hemodialysis or taking n-3 PUFA supplementation. Patients with recent myocardial infarction (onset within 3 months), ongoing congestive heart failure, severe liver dysfunction, or other systemic diseases, including malignancy and collagen disease, were also excluded because these states are known to affect patient characteristics, in particular, parameters related to inflammation. PMI was clinically diagnosed as previously reported [18]. In brief, patients with pathological Q waves on electrocardiography and a region of loss of viable myocardium identified by imaging were considered to have PMI irrespectively of presence of symptoms. In addition, time from the onset of myocardial infarction was at least 3 months in patients with PMI in this study. Patients were defined as having diabetes mellitus (DM) if their hemoglobin A1c (HbA1c) levels exceeded 6.5% or if they were receiving anti-diabetic agents including insulin, regardless of fasting glucose levels [19]. Patients with systolic/diastolic blood pressure >140/90 mmHg on serial measurements [20] and those undergoing treatment with antihypertensive agents were considered to have hypertension. Similarly, patients were diagnosed as having dyslipidemia if they fulfilled the criteria established by the Japan Atherosclerosis Society and other guidelines [21]. We also evaluated the use of the following medications: statins, antiplatelet agents, angiotensin-converting enzyme inhibitors, angiotensin II receptor blockers, calcium channel blockers, beta blockers, and hypoglycemic agents. This study was approved by the Institutional Ethics Committee of each hospital, and all subjects gave informed consent.

Fasting blood samples were obtained in the morning, and serum levels of EPA, DHA, AA, and dihomo-γ-linolenic acid (DHLA) were measured by gas chromatography at an external laboratory (SRL, Inc., Tokyo, Japan). We also evaluated the following laboratory parameters: fasting triglycerides, low-density lipoprotein cholesterol (LDL-C), high-density lipoprotein cholesterol, fasting plasma glucose, HbA1c (Japan Diabetes Society), uric acid, serum creatinine (Cr), and high-sensitivity C-reactive protein (hs-CRP) levels, estimated glomerular filtration rate (eGFR), and brain natriuretic peptide level. eGFR was calculated based on the following Japanese equation, which utilizes Cr level, age, and sex: eGFR (ml/min/1.73 m^2^) = 194 × Cr − 1.094 × age − 0.287 (female × 0.739) [22].

We determined risk factors for PMI using multivariate logistic regression analysis. EPA/AA and DHA to AA ratio (DHA/AA) were tested for associations with each coronary risk factor, such as hypertension, dyslipidemia, and DM, in patients with and without PMI. Ratios of EPA/AA and DHA/AA were evaluated for correlations with HbA1c levels in DM patients with PMI. Finally, effects of statins on PUFAs levels were assessed because statins have been reported to reduce n-3/n-6 PUFA ratios [23].

### Data analysis and statistics

Continuous variables are presented as means ± standard deviations. Categorical variables are presented as percentages. The Student *t* test or Mann–Whitney test was used to compare two groups. The known risk factors (sex, age, DM, hypertension, dyslipidemia, smoking, family history of coronary artery disease (CAD), body mass index (BMI), eGFR, hs-CRP, and (EPA + DHA)/(AA + DHLA) ratio) were selected for univariate and multivariate logistic regression analysis, which was performed to identify factors that correlated with PMI. Variables yielding *P* values <0.10 in univariate analysis were entered into multivariate analysis, except when multicollinearity with selected variables was suspected. Moreover, drugs that were used for secondary prevention were excluded from the multivariate analysis. Patients with missing data for any variable were excluded from the multivariate analysis. Independent factors for EPA/AA and DHA/AA were evaluated in patients with diabetes using the least squares method. The calculations were performed using JMP, version 11.2 (SAS Institute Inc., Cary, NC, USA). A two-sided *P*-value <0.05 was considered to indicate statistical significance.

## Results

**Fig. 1:**
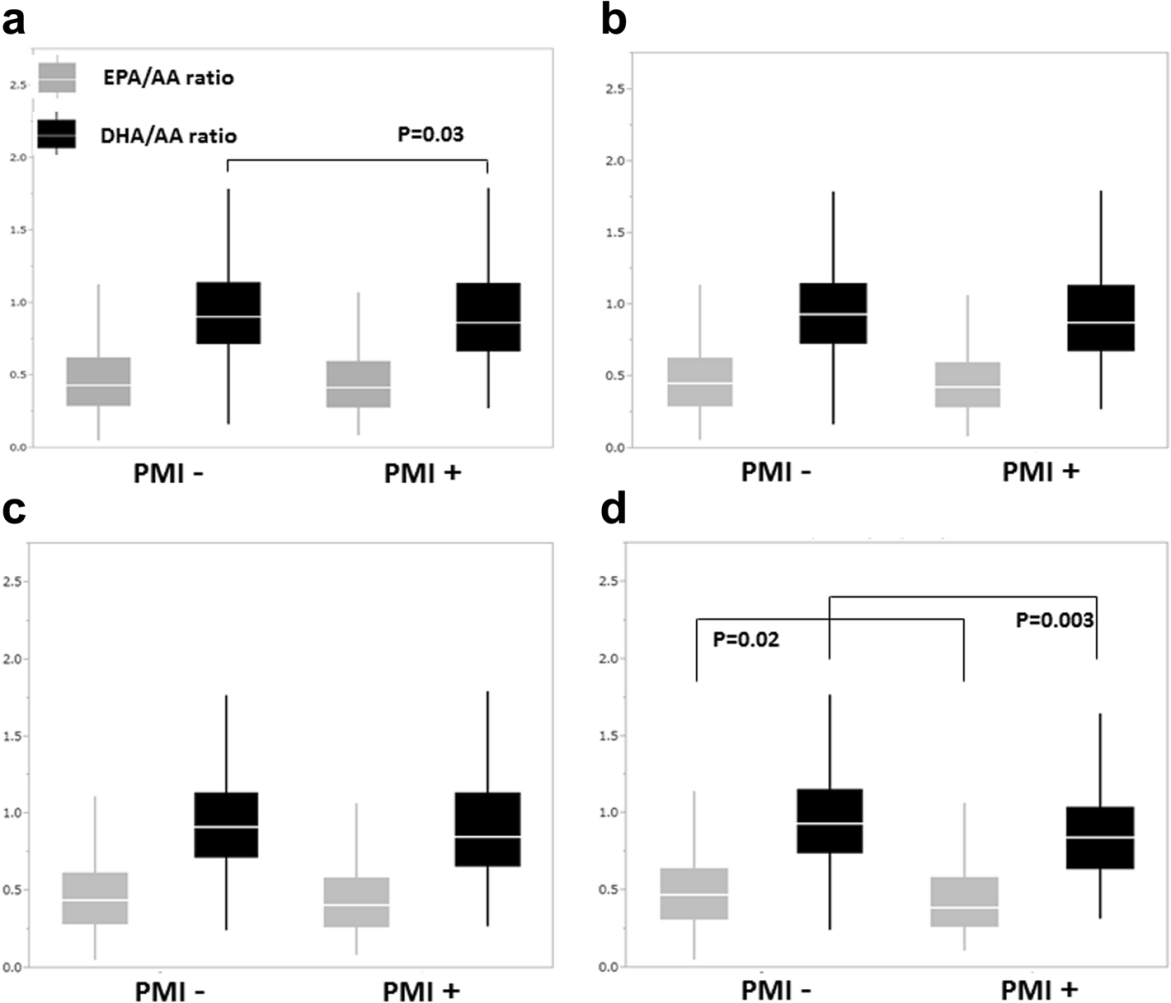
Comparison of ratios of eicosapentaenoic acid (EPA) to arachidonic acid (AA) levels (EPA/AA) and docosahexaenoic acid (DHA) to arachidonic acid (AA) levels (DHA/AA) between the patients with and without prior myocardial infarction (PMI). Analysis for all patients (**a**) and patients with hypertension (HTN; **b**), dyslipidemia (DL; **c**), and diabetes mellitus (DM; **d**). A statistically significant difference in EPA/AA ratios was only present for the patients with DM, whereas that in DHA/AA ratios was present for all and DM patients

**Fig. 2:**
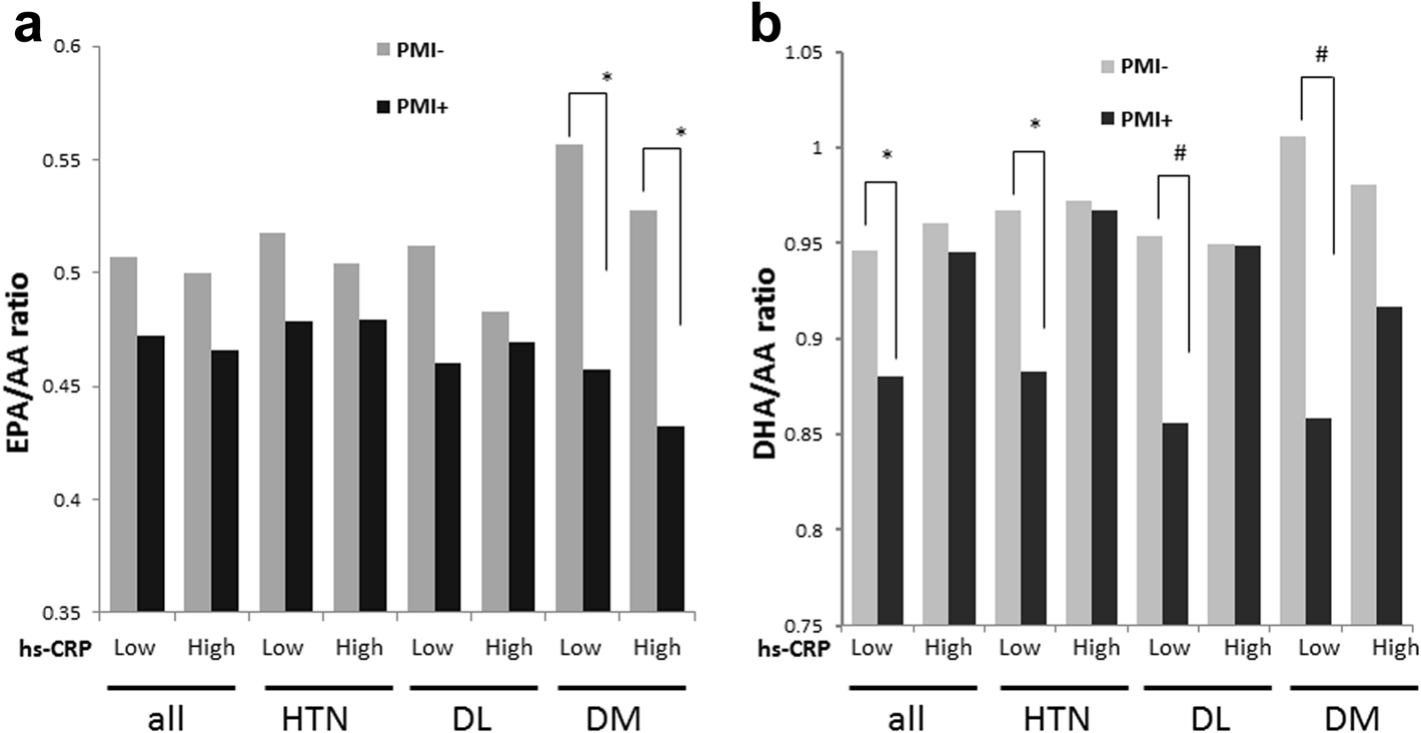
Eicosapentaenoic acid to arachidonic acid (EPA/AA, panel **a**) and docosahexaenoic acid to AA (DHA/AA, panel **b**) ratios were categorized according to high-sensitivity C-reactive protein (hs-CRP) levels (the threshold between low and high hs-CRP levels was set to 0.1 mg/dL) in all, hypertension, dyslipidemia, and diabetes mellitus (DM) patients with or without prior myocardial infarction (PMI). ^*^ *P* < 0.05 (with vs. without PMI); ^#^ *P* < 0.01 (with vs. without PMI)

The clinical backgrounds of the patients are shown in Table 1. Patients with PMI were significantly more often men and smokers and had DM, dyslipidemia, and family history of CAD. Diabetes prevalence in the PMI group was slightly higher than that in a previously described cohort [24]. Statins, beta blockers, and renin angiotensin system blockers were also used more often for secondary prevention in PMI patients; in particular, over 70% of them were on statins. Accordingly, LDL-C levels were lower in patients with PMI. Multivariate logistic regression analysis of PMI prevalence was performed to identify the associated factors (Table 2). Independently associated factors for PMI in this cohort were as follows: male sex, DM, dyslipidemia, family history of CAD, and low (EPA + DHA)/(AA + DHLA) ratio. Since the levels of EPA and DHA were significantly lower in patients with PMI, we investigated whether the EPA/AA and DHA/AA ratios were associated with coronary risk factors such as hypertension, dyslipidemia, and DM in patients with and without PMI (Fig. 1). Although sex and familial history of CAD need to be accounted for as associated factors for PMI, we excluded these factors from evaluation of PUFAs because they were non-modifiable. The EPA/AA ratio was lower in patients with PMI than in patients without PMI, but the difference was not statistically significant. Analysis of subgroups with hypertension, dyslipidemia, and DM revealed that only in patients with DM there was a significant difference in EPA/AA ratios between those with and without PMI (DM patients with PMI: 0.45 ± 0.26; DM patients without PMI: 0.52 ± 0.29; *P* < 0.01). Similarly, DHA/AA levels were significantly lower in patients with PMI, and a significant difference in DHA/AA ratios between those with and without PMI was only observed in the DM subgroup (DM patients with PMI: 0.88 ± 0.32; DM patients without PMI: 0.97 ± 0.33; *P* = 0.003). Additionally, EPA/AA and DHA/AA were evaluated for association with presence of PMI according to BMI and smoking status as other cardiovascular risk factors (Additional file 1: Figure S1). However, in these subgroups, (EPA + DHA)/(AA + DHLA) levels did not differ between PMI and non-PMI patients. Since both DM and low (EPA + DHA)/(AA + DHLA) ratios were associated with PMI, and DM patients with PMI had low levels of EPA and DHA, we compared the background characteristics of patients with each risk factor (hypertension, dyslipidemia and DM) with and without PMI (Table 3). The hypertension and dyslipidemia subgroups had significant differences in numbers of male patients depending on presence of PMI, whereas there was no such difference in the DM subgroup. Although there were no significant differences in hs-CRP levels depending on presence of PMI in these subgroups, levels of hs-CRP tended to be higher in the DM cohort than in the hypertension and dyslipidemia cohorts. Furthermore, the rate of statin use was significantly higher in patients with PMI than in those without PMI, with the highest rate observed in the dyslipidemia cohort. Based on the low EPA/AA and DHA/AA, (EPA + DHA)/(AA + DHLA) ratio was significantly lower in DM patients with PMI than in those without PMI, although this difference was not observed in hypertension and dyslipidemia patients. A previous study showed that patients with high CRP levels had lower EPA/AA ratios [25]; therefore, we analyzed ratios of n-3 PUFAs to AA in patients with different hs-CRP levels (the median hs-CRP level was 0.1 mg/dL). Among those with hs-CRP levels >0.1 mg/L, DM patients with PMI had lower EPA/AA ratios than DM patients without PMI (EPA/AA: DM + high hs-CRP with PMI vs. DM + high hs-CRP without PMI = 0.43 ± 0.24 vs. 0.53 ± 0.30, *P* < 0.05), whereas the DHA/AA ratios did not differ significantly between DM patients with PMI and without PMI in the high hs-CRP cohort (Fig. 2). In contrast to EPA/AA, DHA/AA ratios were significantly lower when corresponding hs-CRP levels were low in hypertension, dyslipidemia, and DM patients with PMI than in hypertension, dyslipidemia, and DM patients without PMI.

**Table 1:** Background characteristics of the patients with and without prior myocardial infarction (PMI)

Variable	PMI patients		*P* value
	+	-	
*N*	303	1430	
Age, y.o	64.5 ± 10.6	64.5 ± 11.5	0.53
Male, *n*	272 (89.8%)	1076 (79.82%)	<0.001
BMI, kg/m^2^	24.4 ± 3.4	24.35 ± 3.5	0.81
HTN, *n*	225 (74.3%)	1042 (72.9%)	0.62
DM, *n*	144 (47.5%)	536 (37.5%)	<0.01
DL, *n*	245 (80.9%)	975 (68.2%)	<0.001
Smoking, *n*	155 (51.2%)	626 (43.8%)	0.02
FH, *n*	70 (23.1%)	251 (17.6%)	0.02
eGFR^a^, ml/min/1.73 m^2^	67.1 ± 19.2	69.01 ± 17.3	0.95
LDL, mg/dL	104.1 ± 29.5	112.4 ± 30.5	<0.001
TGL, mg/dL	147.3 ± 87.8	146.7 ± 97.2	0.53
HbA1c^b^, %	6.0 ± 1.2	5.8 ± 1.1	0.06
UA^c^, mg/dL	6.1 ± 1.4	5.8 ± 1.4	0.97
BNP^d^, pg/mL	85.1 ± 156.3	49.1 ± 73.3	<0.001
hs-CRP, mg/dL	0.23 ± 0.6	0.26 ± 0.8	0.63
Statin, *n*	216 (71.3%)	704 (49.2%)	<0.001
BB, *n*	141 (46.5%)	528 (36.9%)	<0.01
ARB or ACEi, *n*	185 (61.1%)	694 (48.5%)	<0.001
EPA, μg/mL	68.6 ± 36.4	74.96 ± 45.41	0.02
DHA, μg/mL	135.6 ± 49.1	145.1 ± 53.4	<0.01
AA, μg/mL	154.4 ± 38.4	158.1 ± 51.6	0.25
DHLA, μg/mL	32.8 ± 11.8	33.3 ± 12.4	0.49
EPA/AA	0.47 ± 0.3	0.50 ± 0.3	0.08
DHA/AA	0.91 ± 0.3	0.96 ± 0.4	0.03
EPA + DHA/AA + DHLA	1.14 ± 0.5	1.19 ± 0.5	0.07

Continuous variables are presented as means ± standard deviations. Categorical variables are presented as percentages

*BMI* body mass index, *HTN* hypertension, *DM* diabetes mellitus, *DL* dyslipidemia, *FH* family history of coronary artery disease, *eGFR* estimated glomerular filtration rate, *LDL-C* low-density lipoprotein cholesterol, *TGL* triglycerides, *HbA1c* hemoglobin A1c, *UA* uremic acid, *BNP* brain natriuretic peptide, *hs-CRP* high-sensitivity C-reactive protein, *BB* beta blockers, *ACE-I* angiotensin converting enzyme inhibitors, *ARB* angiotensin receptor blockers, *EPA* eicosapentaenoic acid, *DHA* docosahexaenoic acid, *AA* arachidonic acid, *DHLA* dihomo-γ-linolenic acid, *EPA/AA* ratio of EPA to AA levels, *DHA/AA* ratio of DHA to AA levels, *PUFA* polyunsaturated fatty acid

^a^numbers of patients were 247 vs. 1123 (with PMI vs. without PMI, respectively), ^b^numbers of patients were 245 vs. 1103 (same as above), ^c^numbers of patients were 110 vs. 616 (same as above), ^d^numbers of patients were 106 vs. 567 (same as above)

**Table 2:** Multivariate logistic regression analysis of independent risk factors for prior myocardial infarction (PMI patients = 232, non-PMI patients = 1035)

	OR	95% CI		VIF	*P* value
Male	3.36	−1.72	−0.75	1.13	<0.0001
Age	0.99	−0.02	0.01	1.43	0.65
HTN	1.03	−0.32	0.37	1.06	0.85
DM	1.40	−0.64	−0.03	1.10	0.03
DL	2.18	−1.16	−0.42	1.06	<0.0001
Smoking	1.02	−0.33	0.29	1.13	0.88
BMI	1.01	−0.04	0.06	1.13	0.67
FH	1.46	−0.74	−0.001	1.03	0.04
eGFR	1.00	−0.004	0.01	1.19	0.24
EPA + DHA/AA + DHLA	1.47	0.07	0.72	1.09	0.02
hs-CRP	1.09	−0.12	0.34	1.03	0.45

Abbreviations are listed in the footnote to Table 1; *VIF* variance inflation factor

**Table 3:** Patients characteristics according to each risk factor with or without prior myocardial infarction (PMI)

	HTN			DL			DM		
	PMI +	PMI-	*P* value	PMI+	PMI-	*P* value	PMI+	PMI-	*P* value
*n*	225	1039		245	973		144	533	
Age, y.o	65.0 ± 10.5	65.7 ± 10.8	0.35	63.7 ± 10.5	64.6 ± 10.9	0.4	64.9 ± 10.1	66.8 ± 10.1	<0.05
Male, *n*	201 (89.3%)	774 (74.5%)	<0.001	217 (88.6%)	731 (75.1%)	<0.0001	125 (86.8%)	433 (80.9%)	0.11
BMI, kg/m^2^	24.8 ± 3.4	24.7 ± 3.6	0.58	24.9 ± 3.5	24.7 ± 3.5	0.45	24.4 ± 3.3	24.8 ± 3.8	0.25
HTN, *n*	-	-		188 (76.7%)	730 (75.0%)	0.62	110 (76.4%)	417 (77.9%)	0.74
DM, *n*	188 (83.6%)	730 (70.3%)	<0.001	-	-		118 (81.9%)	410 (76.6%)	0.21
DL, *n*	110 (48.9%)	417 (40.1%)	0.02	118 (48.2%)	410 (42.1%)	0.1	-	-	
Smoking, *n*	106 (47.1%)	448 (43.1%)	0.3	117 (47.8%)	454 (46.7%)	0.77	72 (50.0%)	268 (50.1%)	0.98
FH, *n*	56 (24.9%)	186 (17.9%)	0.016	54 (22.0%)	188 (19.3%)	0.37	36 (25.0%)	117 (21.9%)	0.43
eGFR^a^, ml/min/1.73 m^2^	66.5 ± 18.2	67.4 ± 17.6	0.49	66.9 ± 19.5	68.3 ± 17.1	0.32	69.5 ± 19.9	68.3 ± 18.6	0.54
LDL, mg/dL	101.4 ± 28.7	110.8 ± 29.7	<0.0001	103.3 ± 30.2	114.0 ± 32.3	<0.0001	103.3 ± 30.3	110.3 ± 29.4	0.02
TGL, mg/dL	151.7 ± 94.9	148.8 ± 89.6	0.69	154.6 ± 93.4	158.7 ± 103.4	0.61	155.3 ± 107.5	151.9 ± 89.2	0.72
HbA1c^b^, %	6.0 ± 1.1	5.9 ± 1.1	0.12	6.0 ± 1.3	5.9 ± 1.1	0.34	6.7 ± 1.4	6.7 ± 1.2	0.76
UA^c^, mg/dL	6.1 ± 1.3	5.9 ± 1.4	0.11	6.1 ± 1.3	5.8 ± 1.4	0.11	6.0 ± 1.5	5.8 ± 1.3	0.37
BNP^d^, pg/mL	78.9 ± 119.3	49.8 ± 69.1	<0.01	83.9 ± 165.8	42.9 ± 54.2	<0.001	75.0 ± 133.2	54.4 ± 65.7	0.16
hs-CRP, mg/dL	0.26 ± 0.64	0.23 ± 0.67	0.59	0.21 ± 0.54	0.25 ± 0.73	0.56	0.31 ± 0.73	0.30 ± 0.91	0.87
Statin, *n*	167 (74.2%)	551 (53.0%)	<0.001	194 (79.2%)	633 (65.1%)	<0.001	111 (77.1%)	314 (58.7%)	<0.0001
BB, *n*	113 (50.2%)	443 (42.6%)	0.04	118 (48.2%)	376 (38.7%)	<0.01	74 (51.4%)	235 (43.9%)	0.13
ARBor ACEi, *n*	158 (70.2%)	628 (60.4%)	<0.01	157 (64.1%)	479 (49.2%)	<0.0001	96 (66.7%)	281 (52.5%)	<0.01
EPA, μg/mL	69.3 ± 36.5	75.1 ± 43.0	0.06	68.4 ± 36.2	76.4 ± 44.6	<0.01	64.8 ± 33.1	75.5 ± 40.6	<0.005
DHA, μg/mL	135.8 ± 47.8	145.8 ± 51.4	<0.01	136.3 ± 49.8	149 ± 54.3	<0.001	128.9 ± 41.1	143.1 ± 50.4	<0.005
AA, μg/mL	153.4 ± 38.3	156.2 ± 40.8	0.35	157.4 ± 39.1	162.7 ± 43.0	0.08	154.4 ± 39.9	154.1 ± 41.3	0.75
DHLA, μg/mL	32.3 ± 11.9	33.5 ± 12.5	0.22	33.7 ± 11.9	34.9 ± 12.6	0.16	32.5 ± 11.6	33.5 ± 12.7	0.43
EPA/AA	0.47 ± 0.27	0.50 ± 0.30	0.19	0.46 ± 0.28	0.49 ± 0.29	0.21	0.45 ± 0.26	0.52 ± 0.29	<0.01
DHA/AA	0.92 ± 0.35	0.97 ± 0.35	0.06	0.91 ± 0.36	0.95 ± 0.34	0.06	0.88 ± 0.32	0.97 ± 0.33	<0.005
EPA + DHA/AA + DHLA	1.15 ± 0.03	1.22 ± 0.02	0.88	1.13 ± 0.49	1.19 ± 0.49	0.08	1.09 ± 0.44	1.22 ± 0.49	<0.005

Continuous variables are presented as means ± standard deviations. Categorical variables are presented as percentages. Abbreviations are listed in the footnote to Table 1. ^a^numbers of patients were 183 vs. 823, 201 vs. 736, and 119 vs. 416 (HTN with vs. without PMI, DL with vs. without PMI, and DM with vs. without PMI, respectively), ^b^numbers of patients were 181 vs. 809, 200 vs. 728, and 118 vs. 418 (same as above), ^c^numbers of patients were 91 vs. 450, 95 vs. 373, and 46 vs. 168 (same as above), ^d^numbers of patients were 87 vs. 412, 90 vs. 334, and 44 vs. 152 (same as above)

**Fig. 3:**
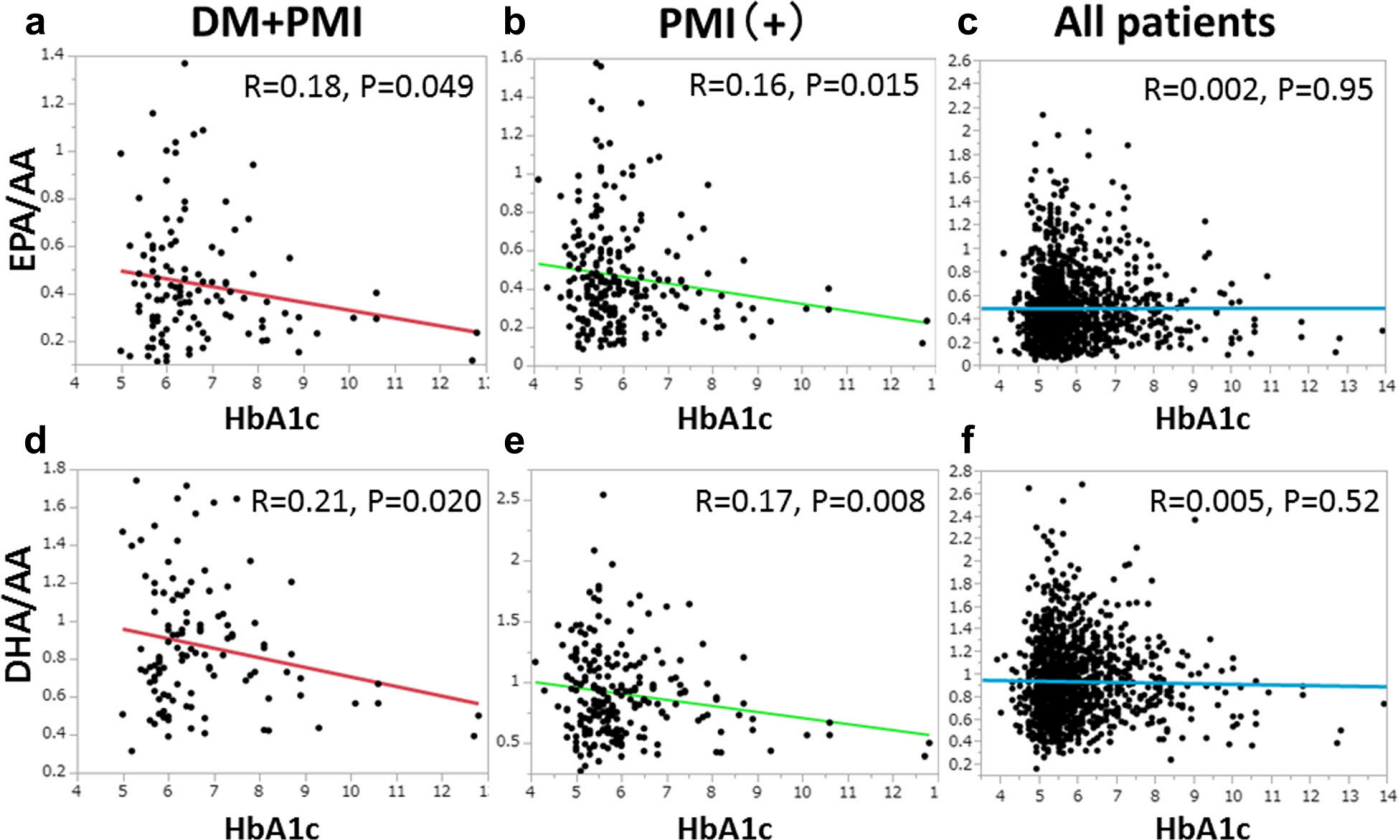
Ratios of n-3 PUFA to AA levels plotted against levels of hemoglobin A1c (HbA1c). The ratio of eicosapentaenoic acid (EPA) to arachidonic acid (AA, EPA/AA) was significantly correlated with HbA1c levels in diabetes mellitus (DM) patients with prior myocardial infarction (PMI) (*N* = 117, *R* = 0.18, *P* = 0.049, panel **a**) and in patients with PMI (*N* = 245, *R* = 0.16, *P* = 0.015, panel **b**), but not in the entire cohort (*N* = 1103, panel **c**). The ratio of docosahexaenoic acid (DHA) to AA (DHA/AA) was significantly correlated with HbA1c levels in DM patients with PMI (*N* = 117, *R* = 0.21, *P* = 0.020, panel **d**) and in patients with PMI (*N* = 245, *R* = 0.17, *P* = 0.008, panel **e**), but not in the entire cohort (*N* = 1103, panel **f**)

Since DM patients with PMI had low n-3 PUFA levels, we evaluated the correlation between levels of HbA1c and ratios of n-3 PUFAs to AA (Fig. 3). Levels of HbA1c were correlated with EPA/AA and DHA/AA ratios in patients with DM and PMI (EPA/AA: *R* = 0.18, *P* = 0.049; DHA/AA: *R* = 0.21, *P* = 0.020), whereas such correlations were not observed in the whole cohort. Moreover, EPA/AA and DHA/AA ratios had a weak correlation with HbA1c level in patients with PMI, but the correlation coefficients were higher in patients with DM and PMI than in patients with PMI. These data suggest that a higher prevalence of uncontrolled diabetes tended to be observed along with lower EPA/AA and DHA/AA in DM patients with PMI. We also evaluated the correlation between levels of HbA1c and ratios of n-3 PUFAs to AA in non-PMI patients; however, there were no correlations in this cohort (Additional file 2: Figure S2).

Because statins have been reported to affect PUFAs levels, associations between statin use and PUFAs levels were evaluated (Table 4). Patients on statins had significantly lower DHA and higher AA and DHLA levels, whereas EPA levels did not depend on statin use. According to these results, DHA/AA was significantly lower in those who received statins. Finally, we analyzed factors independently associated with low EPA/AA and DHA/AA in DM patients (Tables 5 and 6). The factors associated with low EPA/AA ratio were age and PMI. In a similar analysis, such factors for DHA/AA ratio were age, PMI, and statin use. These data indicate that statin use significantly affected DHA/AA levels in DM patients.

**Table 4:** Polyunsaturated fatty acids levels in all patients according to statin use

	Statin use		*P* value
	-	+	
*N*	813	920	
EPA, μg/mL	73.0 ± 43.9	74.6 ± 44.1	0.46
DHA, μg/mL	146.8 ± 57.4	140.5 ± 48.1	<0.05
AA, μg/mL	154.2 ± 57.6	160.3 ± 40.9	<0.01
DHLA, μg/mL	32.2 ± 12.8	34.1 ± 11.8	<0.05
EPA/AA	0.50 ± 0.30	0.49 ± 0.31	0.6
DHA/AA	0.99 ± 0.44	0.91 ± 0.32	<0.0001

Fatty acids (EPA, DHA, AA and DHLA) profile with or without statin use in all study patients

Abbreviations are listed in the footnote to Table 1

**Table 5:** Analysis of independent factors for EPA/AA in patients with diabetes using least squares method

	Estimated value	95% CI		VIF	*P* value
Male	0.050	−0.02	0.12	1.15	0.16
Age	0.004	0.001	0.007	1.44	0.007
HTN	0.026	−0.03	0.09	1.07	0.39
DL	0.011	−0.05	0.08	1.31	0.75
Smoking	−0.016	−0.07	0.04	1.13	0.54
BMI	−0.006	−0.01	0.001	1.27	0.10
FH	0.036	−0.03	0.10	1.03	0.28
PMI	0.042	0.01	0.07	1.07	0.008
eGFR	0.001	−0.0003	0.003	1.19	0.13
Statin use	−0.059	−0.12	0.002	1.29	0.06
hs-CRP	−0.024	−0.05	0.003	1.03	0.08

Abbreviations are listed in the footnote to Tables 1 and 2

**Table 6:** Analysis of independent factors for DHA/AA in patients with diabetes using least squares method

	Estimated value	95% CI		VIF	*P* value
Male	0.014	−0.07	0.09	1.15	0.73
Age	0.006	0.003	0.01	1.44	0.0005
HTN	0.023	−0.05	0.09	1.07	0.50
DL	0.062	−0.02	0.14	1.31	0.13
Smoking	0.043	−0.02	0.11	1.13	0.17
BMI	−0.005	−0.01	0.004	1.27	0.28
FH	0.039	−0.04	0.11	1.03	0.30
PMI	0.037	0.002	0.07	1.07	0.03
eGFR	0.0003	−0.001	0.002	1.19	0.69
Statin use	−0.178	−0.25	−0.11	1.29	<0.0001
hs-CRP	−0.027	−0.06	0.003	1.03	0.08

Abbreviations are listed in the footnote to Tables 1 and 2

## Discussion

The main finding of the present analysis is that PMI patients with DM had lower EPA/AA and DHA/AA ratios, whereas PMI patients with dyslipidemia or hypertension did not. When stratified according to level of hs-CRP, the EPA/AA and DHA/AA ratios behaved differently in patients with and without PMI. Moreover, in DM patients with PMI, there was a weak but significant correlation between the PUFAs to AA ratio and levels of HbA1c. These data suggest that n-3 and n-6 PUFA levels should be monitored in DM patients with PMI, especially in those with severe DM as reflected by a high HbA1c level. In agreement with our results, a previous report showed that DM patients had a low ratio of erythrocyte n-3 PUFAs to fatty acids [26]. The duration of the disease tends to be long in diabetes patients with PMI, with severe chronic inflammation mediated by cytokines [27] accompanied with oxidative stress [28] and endothelial dysfunction [29]. EPA and DHA are known anti-inflammatory agents; in addition, EPA and AA are competing substrates of cyclooxygenase enzymes [16]. Furthermore, metabolites of DHA and EPA have a potent anti-inflammatory effect [30, 31]. More severe inflammation in patients with DM than in patients with dyslipidemia or hypertension may be the reason for the observed association between low levels of n-3 PUFAs and PMI in individuals with DM. Elevated plasma levels of CRP are independently associated with increased risk of atherosclerosis [32, 33], thus hs-CRP is probably one of the most promising biomarkers of vascular inflammation in high-risk patients. A recent cohort study showed that a lower EPA/AA ratio was associated with a greater risk of cardiovascular events in patients with higher hs-CRP levels, whereas no significant association was observed in those with lower hs-CRP levels [25]. These results suggested that cardiovascular events might be influenced by EPA levels in patients with high inflammation. In the present cohort, PMI patients with DM had higher hs-CRP levels than those without hypertension and dyslipidemia, and these patients with high hs-CRP levels had significantly lower EPA/AA ratios than hypertension and dyslipidemia patients with high hs-CRP. We therefore attempted to find a correlation between levels of n-3 PUFAs and hs-CRP; however, no significant correlations were found (data not shown). These results might indicate that a low EPA/AA ratio represents less intense vascular inflammation due to DM than a high hs-CRP level. However, further studies are needed to explore the relationship between vascular inflammation and low EPA/AA ratio in PMI patients with DM.

Previous studies showed that n-3 PUFA supplementation did not prevent adverse cardiac events in PMI patients with DM [9], and a meta-analysis of results of n-3 PUFA intake failed to demonstrate a preventive effect on cardiovascular events [8]. This has created controversy over the use of n-3 PUFAs for secondary prevention, even though the above studies did not evaluate baseline PUFA levels. It was reported that patients with acute coronary syndrome have a low n-3 PUFA level [13], and the incidence of adverse cardiac events in patients who had undergone percutaneous coronary intervention was associated with low EPA/AA ratio [34]. Thus, a low EPA/AA ratio can be potentially used to identify high-risk patients. Another study showed that a higher intake of n-3 PUFAs was associated with a reduced risk of acute myocardial infarction in DM patients [35]. The authors speculated that one of the reasons for the contradicting results was that the subjects in their cohort had higher HbA1c levels than did subjects in the previous report. This conclusion is consistent with our data. It is important to identify groups of patients who can benefit from n-3 PUFA supplementation. One such group may be DM patients with higher HbA1c levels, who might need n-3 PUFA supplementation to reduce the possibility of adverse cardiac events, especially if their CRP level is high.

A previous study showed that patients with a higher EPA/AA, but not DHA/AA, ratio had significantly fewer cardiac events [34]. Similarly, our previous investigation revealed that only a low EPA/AA ratio was significantly associated with acute coronary syndrome [13], and we speculated that one of the reasons might be the differences between EPA and DHA in terms of incorporation into cells or organs [36, 37]. In agreement, a prospective observational study showed that lower plasma EPA, but not DHA, levels were significantly associated with all-cause mortality in patients with acute myocardial infarction [38]. In the present study, EPA/AA ratio behaved differently from DHA/AA ratio in patients with different hs-CRP levels. Thus, DHA/AA ratios were not significantly different in high hs-CRP patients with and without PMI, whereas DHA/AA ratios in patients with low hs-CRP were lower in the presence of PMI. Although both EPA and DHA play important roles in the regulation of inflammation, we surmise that a low EPA/AA ratio, unlike a low DHA/AA ratio, might be related to ongoing vascular inflammation leading to cardiac events in DM patients with PMI. On the other hand, the finding that patients with PMI had a high rate of statin use in our cohort is compatible with preventing secondary cardiovascular events. Statins have been reported to affect n-3 and n-6 PUFA levels [23], and our data showed that statin use is significantly associated with low DHA and high AA and DHLA levels, but not with EPA levels. A previous report showed that statins decreased serum DHA levels and the DHA/AA ratio without altering the EPA/AA ratio [39]. These results are in agreement with our data, and it seems that statins littlle affect EPA/AA ratio in DM patients with PMI, whereas they might lower the DHA/AA ratio in these patients. Since statins also exert an anti-inflammatory effect by lowering the hs-CRP levels, we speculate that statin use might be a cause of the low DHA/AA ratio in low hs-CRP patients with PMI. Based on these considerations, EPA/AA ratio is more suitable than DHA/AA ratio for evaluation of the PUFA profile in clinical practice.

Our data showed that the decreased EPA/AA ratio was mainly caused by low EPA levels and not by AA levels. A previous report suggested that high AA levels correlated with low probability of cardiac events [40]; therefore, high serum AA levels do not always have a negative impact in cardiovascular disease. Although AA has an important positive role in inflammation, which can be attributed to infection and wound healing, the nature of inflammation differs between patients with a chronic disease like atherosclerosis and those with nonspecific inflammatory processes. Furthermore, there has been no interventional study of AA supplementation, whereas n-3 supplementation has been reported to have at least a partial protective effect with respect to cardiovascular events [3–5].

The present study has some limitations. First, because of the retrospective and observational nature of the analysis, a possibility of selection bias may not be entirely excluded. Owing to the stringent inclusion criteria, the number of subjects was relatively small. Hence, a large-scale, multi-center, prospective study is necessary to confirm the results of this report. Second, since this was a cross-sectional study, a causal relationship between diabetes and imbalance of PUFAs profile could not be verified. Third, although the ratio of n-3 PUFAs to total PUFAs levels may be a better indicator, we evaluated only 4 PUFAs in this study, because these PUFAs are involved in inflammation related to cardiovascular disease. Although the mol% ratio of n-3 PUFAs to total free fatty acids is widely used, for the most part it reflects the nutritional status. The present urban Japanese cohort has a relatively uniform nutritional status (malnutrition was uncommon), and therefore we did not evaluate the mol% ratio of n-3 PUFAs to total free fatty acids. Despite these limitations, we suggest that PMI patients with DM have lower EPA/AA ratios.

## Conclusion

PMI patients with DM, but not hypertension or dyslipidemia, have lower EPA/AA and DHA/AA ratios. The additional association found for DM patients with PMI suggests that EPA/AA and DHA/AA behave differently in individuals with different hs-CRP levels. Statins can potentially affect DHA/AA but not EPA/AA, and therefore EPA/AA is a better marker of assessment for cardiovascular events. Among patients with PMI, those with diabetes and high hs-CRP levels may benefit from PUFA supplementation.

## Additional files

Additional file 1: Figure S1. Comparison of ratios of eicosapentaenoic acid (EPA) to arachidonic acid (AA) levels (EPA/AA) and docosahexaenoic acid (DHA) to arachidonic acid (AA) levels (DHA/AA) between the patients with and without prior myocardial infarction (PMI). Panel A shows the results of analysis of smoking status, and Panel B shows the results of analysis of body mass index (BMI). B1 and B2 indicate EPA/AA with and without PMI plotted against levels of BMI, respectively. B3 and B4 indicate DHA/AA with and without PMI plotted against levels of BMI, respectively. There were no significant differences or correlations between EPA and DHA/AA and the examined factors. (TIF 145 kb)
Additional file 2: Figure S2. Panel A shows the ratio of eicosapentaenoic acid (EPA) to arachidonic acid (AA, EPA/AA) plotted against levels of hemoglobin A1c (HbA1c) in patients without PMI. Panel B shows the ratio of docosahexaenoic acid (DHA) to AA (DHA/AA) plotted against levels of HbA1c in patients without PMI. (TIF 115 kb)

## Abbreviations

AA: Arachidonic acid
BMI: Body mass index
CAD: Coronary artery disease
Cr: Serum creatinine
DHA: Docosahexaenoic acid
DHA/AA: Ratio of serum levels of DHA to arachidonic acid
DM: Diabetes mellitus
eGFR: Estimated glomerular filtration rate
EPA: Eicosapentaenoic acid
EPA/AA: Ratio of serum levels of EPA to arachidonic acid
HbA1c: Hemoglobin A1c
hs-CRP: High-sensitivity C-reactive protein
LDL-cholesterol: Low-density lipoprotein cholesterol
PMI: Prior myocardial infarction
PUFA: Polyunsaturated fatty acid
