# Supplementary figures and images for: The ratio of serum n-3 to n-6 polyunsaturated fatty acids is associated with diabetes mellitus in patients with prior myocardial infarction: a multicenter cross-sectional study
Source: BMC Cardiovasc Disord. 2017 Jan 26;17:41. doi: 10.1186/s12872-017-0479-4 (PMC5270364; doi:10.1186/s12872-017-0479-4)

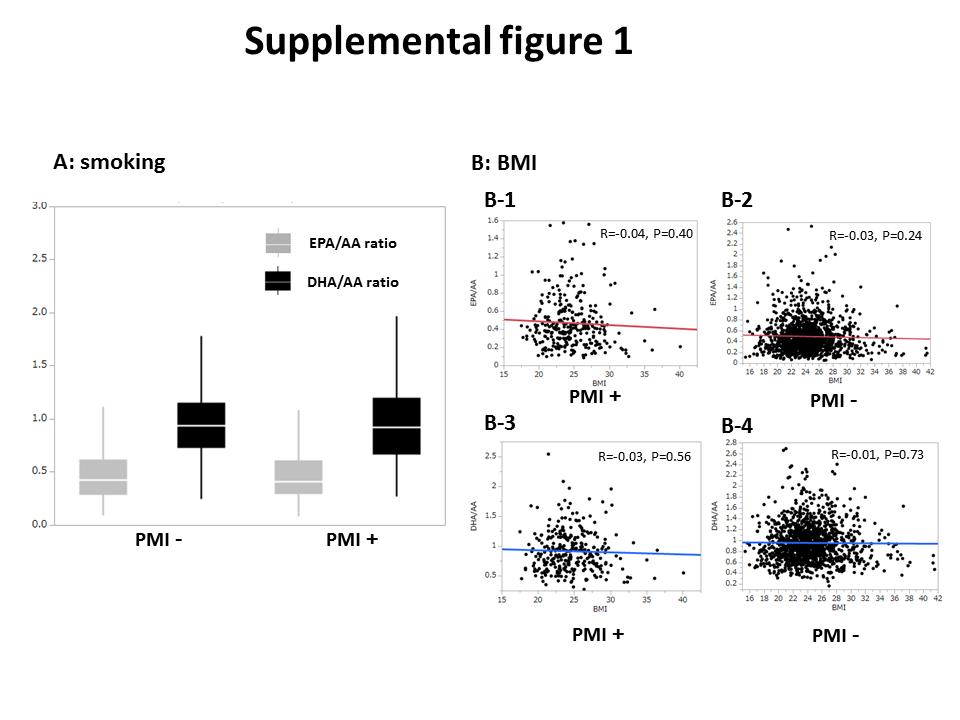

Supplement: Additional file 1: Figure S1. — Comparison of ratios of eicosapentaenoic acid (EPA) to arachidonic acid (AA) levels (EPA/AA) and docosahexaenoic acid (DHA) to arachidonic acid (AA) levels (DHA/AA) between the patients with and without prior myocardial infarction (PMI). Panel A shows the results of analysis of smoking status, and Panel B shows the results of analysis of body mass index (BMI). B1 and B2 indicate EPA/AA with and without PMI plotted against levels of BMI, respectively. B3 and B4 indicate DHA/AA with and without PMI plotted against levels of BMI, respectively. There were no significant differences or correlations between EPA and DHA/AA and the examined factors. (TIF 145 kb) [file 12872_2017_479_MOESM1_ESM.tif]

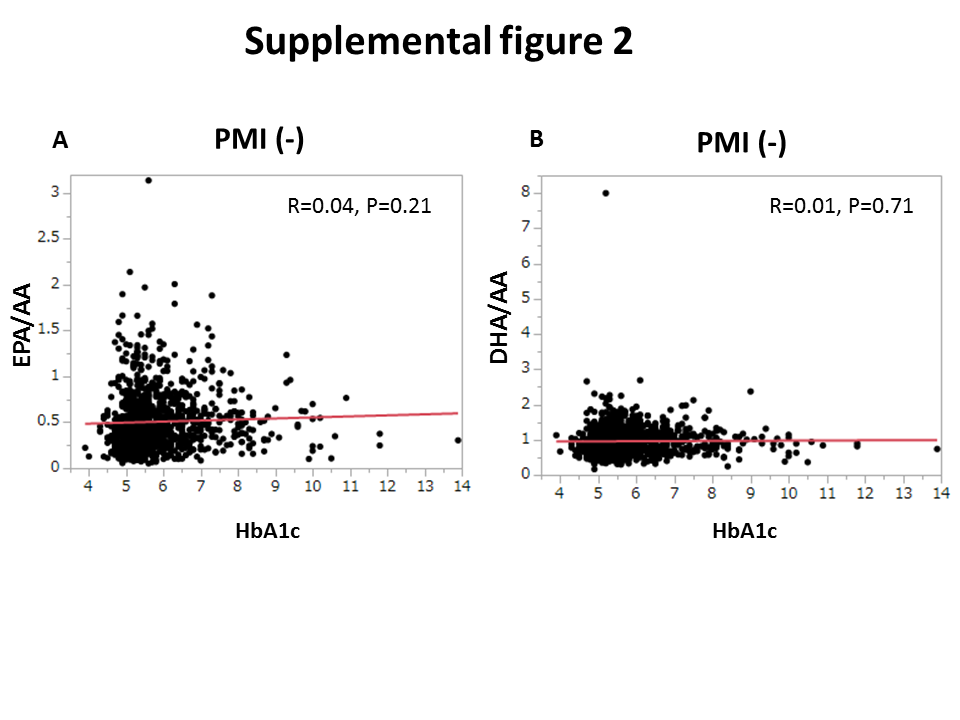

Supplement: Additional file 2: Figure S2. — Panel A shows the ratio of eicosapentaenoic acid (EPA) to arachidonic acid (AA, EPA/AA) plotted against levels of hemoglobin A1c (HbA1c) in patients without PMI. Panel B shows the ratio of docosahexaenoic acid (DHA) to AA (DHA/AA) plotted against levels of HbA1c in patients without PMI. (TIF 115 kb) [file 12872_2017_479_MOESM2_ESM.tif]
